# Nonorgan-specific autoantibodies in HIV-infected patients in the HAART era

**DOI:** 10.1097/MD.0000000000006230

**Published:** 2017-03-10

**Authors:** Laura Iordache, Djaouida Bengoufa, Olivier Taulera, Agathe Rami, Caroline Lascoux-Combe, Nesrine Day, Maguy Parrinello, Pierre-Olivier Sellier, Jean-Michel Molina, Alfred Mahr

**Affiliations:** a Department of Internal Medicine, Saint Louis Hospital, University Paris Diderot; b Department of Immunology and Histocompatibility, Saint Louis Hospital, University Paris Diderot; c Department of Internal Medicine, Lariboisière Hospital, University Paris Diderot; d Department of Infectious Diseases, Saint Louis Hospital, University Paris Diderot; e Chemin Vert Biology Center, Paris, France.

**Keywords:** ANCAs, antinuclear antibodies, autoantibodies, HAART, HIV, hypergammaglobulinemia

## Abstract

Nonorgan-specific autoantibodies (AAbs) are used for diagnosing autoimmune diseases but can also be detected in other conditions. We carried out a cross-sectional study with the aim to screen HIV1-infected patients in the era of highly active antiretroviral therapy (HAART) for AAbs and to analyze the association of their presence with hypergammaglobulinemia and immunovirological status.

Blood samples from HIV1-infected patients without major concomitant illnesses followed in 2 hospitals in Paris, France were tested for immunovirological status, serum immunoglobulin G (IgG) level, antinuclear antibodies (ANAs), anti-double-stranded DNA (anti-dsDNA), anti-extractable nuclear antigens (anti-ENAs), anticardiolipin (aCL), anti-β2glycoprotein1 (anti-β2GP1), and antineutrophil cytoplasmic antibodies (ANCAs). Clinically relevant AAbs were defined as ANAs with titers ≥1:160, anti-dsDNA or anti-ENA antibodies; aCL or anti-β2GP1 antibodies with a level ≥40 U/ml; and ANCAs reacting with proteinase 3 or myeloperoxidase.

We included 92 patients (mean age 47 years, men 55%, sub-Saharan African background 55%, HAART 85%, mean CD4 lymphocyte count 611/mm^3^, viral load < 40 copies/mL 74%). At least 1 AAb was detected in 45% of patients, mostly ANAs (33%) and ANCAs (13%); 12% had ≥1 clinically relevant AAb. Above-normal IgG levels were found in 71% of patients. We found an inverse association between the presence of ≥1 AAb and CD4 lymphocyte count (*P* = 0.03) and between above-normal IgG levels and duration of virological control (*P* = 0.02) and non-sub-Saharan African background (*P* = 0.001).

In sum, in HIV1-infected patients without any major concomitant illness in the HAART era, the prevalence of AAbs remains high but AAb patterns leading to high suspicion of autoimmune diseases are rather uncommon. AAb presence is associated with reduced CD4 lymphocyte count but not hypergammaglobulinemia.

## 1 Introduction

Antinuclear antibodies (ANAs), antiphospholipid (aPL) and antineutrophil cytoplasmic antibodies (ANCAs) are nonorgan-specific autoantibodies (AAbs) tightly linked with autoimmune diseases, namely connective tissue disorders, antiphospholipid syndrome, and ANCA-associated vasculitis. The diagnostic relevance increases for ANAs with titers ≥1:160 and specifically reacting with extractable nuclear antigens (ENAs) or double-stranded DNA (dsDNA)^[1]^; for aPL with anticardiolipin (aCL) or anti-β2glycoprotein1 (anti-β2GP1) specificities^[2]^ and levels ≥40 IgM phospholipid units (MPL)/mL, IgG phospholipid units (GPL)/mL or U/mL; and for ANCAs reacting with proteinase 3 (PR3) or myeloperoxidase (MPO).^[3]^ AAbs may also be found in healthy people^[4,5]^ or in other conditions such as infections^[6]^ or cancers.^[7]^ In clinical practice, such “false-positive” test results can lead to unnecessary further testing for suspected autoimmune diseases and erroneous treatment decisions.

Earlier studies detected AAbs in 20% to 45% of HIV-infected patients.^[8]^ The mechanisms potentially involved in the production of AAbs in the context of HIV infection include molecular mimicry,^[9,10]^ dysregulation of the interaction between B and T lymphocytes,^[11]^ and polyclonal B lymphocyte activation. Studies investigating this latter hypothesis did not find a significant relationship between polyclonal hypergammaglobulinemia and the presence of ANAs, ANCAs, or aPL, but these studies were mostly performed in the pre-highly active antiretroviral therapy (HAART) era and patients’ immunovirological status was not always well described.^[8,12–15]^ So far, little is known about the presence and predictors of such AAbs in asymptomatic HIV-infected patients on HAART.

The aim of this research was to study the prevalence of nonorgan-specific AAbs in HIV1-infected patients in the HAART era and to analyze whether their presence is associated with hypergammaglobulinemia or HIV control.

## 2 Methods

### 2.1 Patient selection

This cross-sectional observational study included patients with known HIV1 infection followed in 2 hospitals in Paris, France. We excluded patients with comorbidities such as chronic viral hepatitis, cancer, autoimmune diseases, or any other active infection. Demographic information and previous history of HIV infection were retrieved from medical records. Geographic background was based on the information available in the medical records and dichotomized in 2 subsets: sub-Saharan African origin and other. All patients provided written informed consent to participate to the study. The study was approved by the local ethics committee (Institutional Review Board No. 00003835).

### 2.2 Laboratory tests

Blood samples were taken during a routine follow-up visit that included the measurement of plasma HIV viral load (VL) and CD4 lymphocyte count. Undetectable VL was defined as <40 copies/mL. AAb analyses were performed in a central laboratory and included tests for ANAs, anti-ENA, anti-dsDNA, aCL, anti-β2GP1 antibodies, and ANCAs. ANAs were detected by an indirect immunofluorescence technique (negative cut-off titer: 1:80); anti-dsDNA antibodies were detected by using the Euroimmun enzyme-linked immunosorbent assay (ELISA) anti-ADN-NcX (IgG) kit (negative cut-off level: 100 U/mL); anti-ENAs were detected by using the Varelisa ANA 8 ELISA Screen kit (detecting anti-centromere CENP-B, anti-JO1, anti-RNP, anti-Scl70, anti-Sm, anti-SSA/Ro, and anti-SSB/La antibodies; negative cut-off ratio: 1.4); aCL and anti-β2GP1 antibodies were detected by using an anti-CL ELISA kit (negative cut-off level: 20 MPL/mL and GPL/mL) and B2GP1 ELISA kit (negative cut-off level: 15 U/mL IgG and IgM) or the Bioplex2200 Biorad immunoassay kit (negative cut-off level: 20 MPL/ml and GPL/mL, 20 U/mL IgG and IgM); ANCAs were detected by indirect immunofluorescence (negative cut-off titer: 1:40) and in case of positivity, specificity was characterized by using the ANCA-Profile ELISA kit (detecting anti-PR3, anti-MPO, anti-elastase, anti-lactoferrin, anti-cathepsin G, and anti-bactericidal/permeability increasing protein [BPI] antibodies). All commercial tests were performed according to the instructions provided by the manufacturers. Serum immunoglobulin G (IgG) level was measured with the Modular P-Roche turbidimetric technique (normal values: 6.7–12.4 g/L). Above-normal IgG levels were defined as values above the upper limit (> 12.4 g/L) of the laboratory norm.

### 2.3 Statistical analyses

Data were expressed as number (%) and mean (standard deviation [SD]) for categorical and continuous variables, respectively. AAb positivity was defined according to the laboratory cut-off (for ANAs) or the cut-offs provided by the manufacturers of the commercial tests. Because in clinical practice some positive AAb tests have little or no diagnostic relevance,^[1–3]^ we defined clinically relevant AAb titers as follows: ANAs with titers ≥1:160; anti-ENA; anti-dsDNA; aCL or anti-β2GP1 antibodies with levels ≥40 MPL/mL, GPL/mL or U/mL; and ANCAs with anti-PR3 or anti-MPO specificity.

We studied several immunovirological characteristics and the geographic background by presence of ≥1 AAb, ≥1 clinically relevant AAb, and above-normal IgG levels. The following variables were analyzed: current absolute and relative CD4 lymphocyte counts, lowest ever CD4 lymphocyte count, time since CD4 lymphocyte count > 200/mm^3^ or >15%, current undetectable VL, highest ever VL, time since an undetectable VL was obtained and sub-Saharan African background. Continuous and categorical variables were compared by Student *t* or Wilcoxon and Chi-Square or Fisher tests, as appropriate. To search for confounding factors among variables found associated with the outcomes analyzed, we used multivariate analyses with logistic regression models.

Analyses involved use of R v2.8.0 (R Foundation for Statistical Computing, Vienna, Austria). Two-sided *P* < 0.05 was considered statistically significant.

## 3 Results

The study included 92 patients between April 2014 and October 2015. The mean (SD) age was 46.7 (10.5) years, 51 (55%) were men and 51/90 (55%) were of sub-Saharan African background (n = 2 not determined). The mean (SD) time since diagnosis of HIV infection was 10.5 (7.9) years. Overall, 78 (85%) patients received HAART. The category of HIV infection by the US Centers for Disease Control and Prevention classification system was A for 68 (74%) patients, B for 4 (4%) patients, and C for 20 (22%) patients. The VL was undetectable in 68 (74%) patients for a mean (SD) duration of 6.5 (4.8) years. The mean (SD) absolute and relative CD4 lymphocyte counts were 611 (302)/mm^3^ and 30% (10%), respectively. The CD4/CD8 lymphocyte ratio was <1 for 64 patients (70%).

In all, 41 patients (45%) had ≥1 AAb, whereas 12 (29%) and 2 (5%) had 2 and 3 AAbs, respectively (Table 1). The most commonly detected AAb types were ANAs in 30 (33%) patients and ANCAs in 12 (13%) patients. Eleven patients (12%) satisfied our definition of ≥1 clinically relevant AAb. A total of 65 patients (71%) had above-normal IgG levels and for 39 (42%) and 23 (25%), IgG levels were ≥15 and ≥17 g/L, respectively.

**Table 1:**
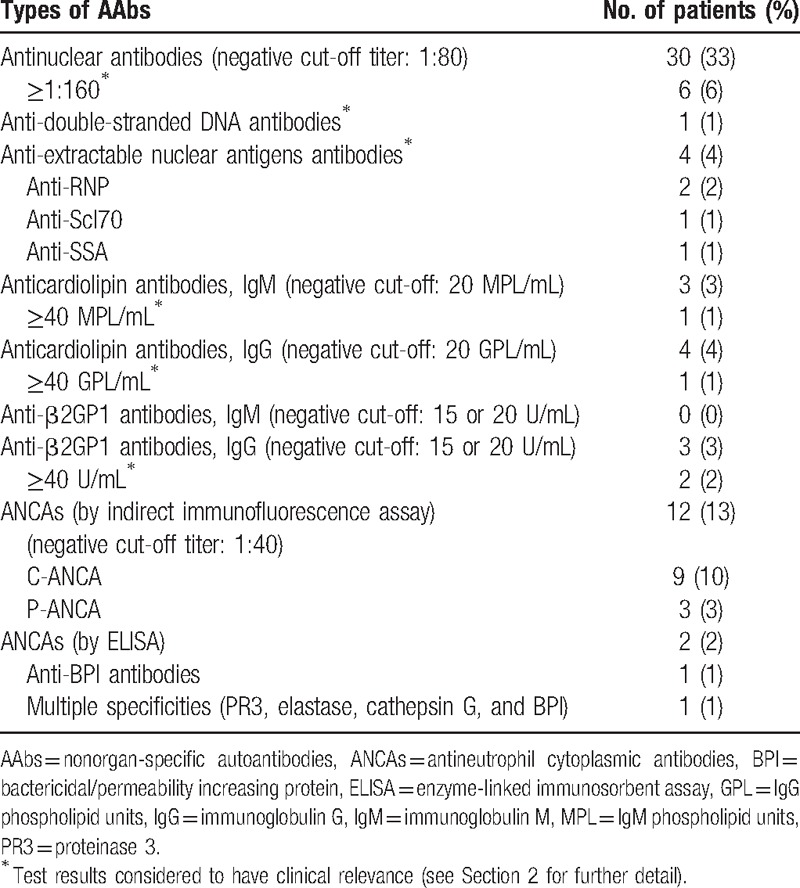
Results of screening for nonorgan-specific autoantibodies in 92 HIV1-infected patients.

For patients with than without ≥1 AAb, mean absolute and relative CD4 lymphocyte counts were lower (*P* = 0.007 and 0.01, respectively) and mean serum IgG levels were higher (*P* = 0.02), but the groups did not differ in percentages of patients with above-normal IgG levels (*P* = 0.17) (Table 2). On multivariate analysis, absolute CD4 lymphocyte counts remained inversely associated with the presence of ≥1 AAb (*P* = 0.03) after adjustment for serum IgG levels (*P* = 0.10). We found no difference between patients with or without clinically relevant AAbs.

**Table 2:**
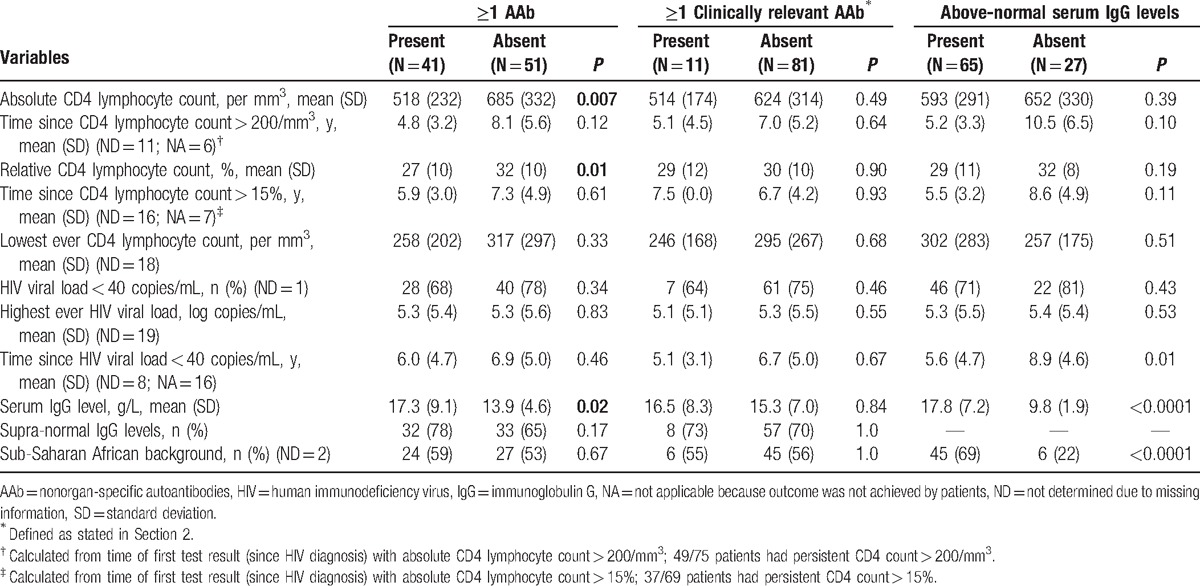
Immunovirological characteristics, serum immunoglobulin G levels, and geographic background in 92 HIV1-infected patients with versus without nonorgan specific autoantibodies, clinically relevant nonorgan specific autoantibodies, and above-normal serum immunoglobulin G levels.

For patients with than without above-normal IgG levels, the time since the VL had become undetectable was shorter (*P* = 0.02) and a sub-Saharan African background more frequent (*P* = 0.001) (Table 2). Both variables remained independently associated with above-normal IgG levels on multivariate analysis (*P* = 0.02 and *P* = 0.001, respectively) and remained unchanged in a sensitivity analysis that excluded 1 patient with an extremely high IgG level (60.1 g/L) (detailed results not shown).

## 4 Discussion

In our study of 92 HIV1-infected patients without concomitant diseases and mostly good viral control and immunological status in the HAART era, we found that 45% had at least 1 AAb, especially ANAs and ANCAs, according to the cut-off values established by the manufacturers. The prevalence of ANAs we estimated is in the same range as that reported from historical control data for HIV-infected patients^[14,16]^ and up to 3 times greater than in healthy people.^[4,5]^ By contrast, the prevalence of ANCAs and aCL seems lower than in historical studies of mostly smaller patient groups at a more advanced disease stage with reported prevalences of 48% for ANCAs^[8]^ and 41% for aCL.^[15]^

Evidence is mounting that viral replication plays a critical role in the synthesis of AAbs in HIV infection. The low prevalence of aCL in our study of patients including a high proportion of HAART recipients with an undetectable VL agrees with a study showing a positive association between the VL and the presence and level of aCL.^[10]^ The view that immune dysregulation regresses with suppression of viral replication by HAART, even though not completely, is further supported by our finding of an association between the presence of AAbs and a less effective immunological control. Our results also align with the results of another study showing a decrease in AAb responses after HAART introduction.^[17]^

The observation that cellular immune reconstitution results in a lower production of autoantibodies provides some insights into how T cells may play a role in autoimmunity. One of the immunological mechanisms suggested to explain the lower frequency of AAbs in HIV patients on HAART than in HAART-naive patients is the observed reduction in CD33^+^CD11b^+^HLA-DR^+^ myeloid-derived suppressor cells,^[17]^ which have a T-cell-suppressing function. Whether the fact that ANAs, which appeared highly prevalent in our patient population despite high coverage with HAART, reflects a role of this AAb as a broadly neutralizing antibody^[18]^ deserves further investigation.

We found no association between the presence of AAbs and polyclonal hypergammaglobulinemia even though 71% of our patients had above-normal serum IgG levels. Therefore, our study reinforces the fact that hypergammaglobulinemia per se does not account for AAb production in HIV infection. Our and other studies indicating that 37% of patients receiving treatment still show elevated serum IgG levels^[19]^ also highlights the complex impact of HAART on B-lymphocyte activation. The finding of a significantly greater proportion of patients of sub-Saharan African background showing above-normal IgG levels agrees with previous studies of healthy^[20]^ and HIV-infected people^[21]^ and might reflect a genetic predisposition or other causes.

From a clinical perspective, ANAs were mostly found with low titers and ANCAs with antigenic specificities of little clinical relevance. Eventually, only a few of the patients we analyzed had clinically relevant AAb profiles that could raise high suspicion for an underlying autoimmune disease (e.g., ANAs with titers ≥1:160 specifically reacting with dsDNA or ENAs; aCL or anti-β2GP1 antibodies with levels ≥40 MPL/mL, GPL/mL or U/mL; ANCAs with anti-PR3 or anti-MPO specificity). Collectively, these results indicate that for HIV-infected patients with good immunovirological status, the detection of nonorgan-specific AAbs with levels or specificities usually considered clinically relevant should not be summarily dismissed as false-positive results. This consideration is of importance because autoimmune diseases may develop in HIV-infected patients who recovered a good immune status.^[16]^

Our study has limitations. We acknowledge its relatively limited sample size, which is nonetheless in the range of that of previous similar studies, and we believe that it allowed for obtaining reliable estimates. The lack of follow-up precludes determining whether an autoimmune disease eventually develops in some patients with AAbs, but this situation would not have altered our main conclusions.

In summary, the prevalence of nonorgan-specific AAbs remains high in the HAART era but may have decreased in patients who achieved a good immunological status, and their presence is not associated with hypergammaglobulinemia. Most importantly, the AAbs we found in this context were generally not clinically relevant, which implies that the diagnostic specificity of these clinically relevant AAbs remains intact in HIV-infected patients. These results should provide guidance to clinicians when prescribing and analyzing results of AAb tests in HIV-infected patients.
